# Convexity subarachnoid hemorrhage revealed contralateral internal carotid artery dissection due to Eagle syndrome: a case report

**DOI:** 10.1186/s12883-024-03890-y

**Published:** 2024-10-08

**Authors:** Kazuki Obara, Takahiro Furuta, Chikako Yagi, Noriyoshi Nakai, Junichiro Suzuki, Masahisa Katsuno, Yasuhiro Ito

**Affiliations:** 1 Department of Neurology, TOYOTA Memorial Hospital, Toyota, Aichi Japan; 2 Department of Neurology, Nagoya University Graduate School of Medicine, Nagoya, Aichi Japan; 3 Clinical Memory Research Unit, Department of Clinical Sciences, Faculty of Medicine, Lund University, Lund, Sweden; 4 Department of Otolaryngology - Head and Neck Surgery, Fujita Health University, Toyoake, Aichi Japan; 5 Department of Clinical Research Education, Nagoya University Graduate School of Medicine, Nagoya, Aichi Japan

**Keywords:** Carotid artery dissection, Eagle syndrome, Convexity subarachnoid hemorrhage, Elongated styloid process, Headache

## Abstract

**Background:**

Atraumatic localized convexity subarachnoid hemorrhage (cSAH) is an uncommon form of nonaneurysmal subarachnoid hemorrhage characterized by bleeding limited to the cerebral convexities. Ipsilateral cSAH can result from a variety of causes, such as internal carotid artery stenosis, obstruction, and dissection, although concomitant contralateral cSAH is exceptionally rare. In this case, the initial findings of cSAH led us to discovering contralateral internal carotid artery dissection (ICAD) and an elongated styloid process (ESP). ESP is recognized as a risk factor for ICAD, which is a hallmark of Eagle syndrome. This sequence of findings led to the diagnosis of Eagle syndrome, illustrating a complex and intriguing interplay between cerebrovascular conditions and anatomical variations.

**Case presentation:**

A 47-year-old Japanese woman experienced acute onset of headache radiating to her neck, reaching its zenith approximately two hours after onset. Given the intractable nature of the headache and its persistence for three days, she presented to the emergency department. Neurological examination revealed no abnormalities, and the coagulation screening parameters were within normal ranges. Brain computed tomography (CT) revealed right parietal cSAH, while CT angiography (CTA) revealed ICAD and an ESP measuring 30.1 mm on the left side, positioned only 1.4 mm from the dissected artery. The unusual occurrence of contralateral cSAH prompted extensive and repeated imaging reviews that excluded reversible cerebral vasoconstriction syndrome (RCVS), leading to a diagnosis of left ICAD secondary to Eagle syndrome. The patient underwent conservative management, and the dissected ICA spontaneously resolved. The patient has remained recurrence-free for two and a half years.

**Conclusions:**

Managing cSAH requires diligent investigation for ICAD, extending beyond its identification to explore underlying causes. Recognizing Eagle syndrome, though rare, as a potential etiology of ICAD necessitates the importance of evaluating ESPs. The method for preventing recurrent cervical artery dissection due to Eagle syndrome is controversial; however, conservative management is a viable option.

## Background

Atraumatic localized convexity subarachnoid hemorrhage (cSAH) is an unusual subtype of nonaneurysmal subarachnoid hemorrhage (SAH) in which bleeding is limited solely to the cerebral convexities. This condition can arise from various causes, including reversible cerebral vasoconstriction syndrome (RCVS), cerebral amyloid angiopathy, and posterior reversible encephalopathy syndrome (PRES), the first of which is the predominant cause among younger individuals [1]. Additionally, internal carotid artery (ICA) stenosis, occlusion, and dissection are recognized as factors associated with cSAH [1–4], typically occurring ipsilateral to the cSAH [4–8]. Contralateral occurrences, though exceedingly rare, have also been documented [9].

An elongated styloid process (ESP) is considered a risk factor for internal carotid artery dissection (ICAD), a recognized vascular subtype of Eagle syndrome [10–12]. Thus, it is theoretically possible that ESP could lead to ICAD, which subsequently cause cSAH. However, documented cases of such sequences are scarce.

In this case, the investigation of a right-sided cSAH unexpectedly revealed a left-sided ICAD, which further led to the discovery of an ESP and ultimately resulted in a diagnosis of Eagle syndrome. This series of discoveries underscores the critical need for an exhaustive and meticulous diagnostic process for patients presenting with cSAH.

## Case presentation

A 47-year-old Japanese woman with a medical background marked by migraine experienced acute onset of pulsatile and throbbing headache radiating to her neck. The patient denied any history of preceding trauma, excessive neck movement, straining on the toilet, or flu-like symptoms. She did not have hypertension, diabetes mellitus, or heart disease. She was not a tobacco or alcohol consumer; her sole regular medication consisted of magnesium oxide for constipation. There was no family history of migraine, stroke, or seizures. She had a history of migraine headache for more than 35 years, with three or four attacks per month. Typically, these headaches responded well to ibuprofen treatment.

During the episode for which she came to our hospital, her headache reached a zenith of 9 out of 10 on the narrative rating scale (NRS) approximately two hours after onset. Assuming it to be another instance of her usual headache, she adhered to her customary practice of taking ibuprofen. However, contrary to her expectations, the pain persisted. Consequently, recognizing the intractable nature of the headache and its persistence for three days, she presented to the emergency department. Other than her persistent headaches, she reported no other discernible neurological abnormalities.

On examination, she was afebrile, with a blood pressure of 151/93 mmHg, pulse rate of 70/min, respiratory rate of 15/min, and oxygen saturation of 100% on room air. Cardiopulmonary, abdominal, and musculoskeletal examinations did not reveal any relevant findings. The neurological examination was normal.

### Testing

Laboratory investigations revealed a normal hemogram, blood sugar, lipid profile, kidney function, liver function, and electrolyte profile. The levels of autoimmune markers, including antinuclear antibody (1:20 dilution), rheumatoid factor (3.4 IU/mL), and anti-SS-A antibody, were unremarkable. Coagulation screening parameters, such as prothrombin time (12.4 s), activated partial thromboplastin time (25.7 s), and D-dimer (0.7 µg/mL), were within normal ranges. Similarly, the levels of inflammatory markers, including C-reactive protein (2.0 mg/L) and erythrocyte sedimentation rate (7 mm at the end of 1 h), were normal.

**Fig. 1:**
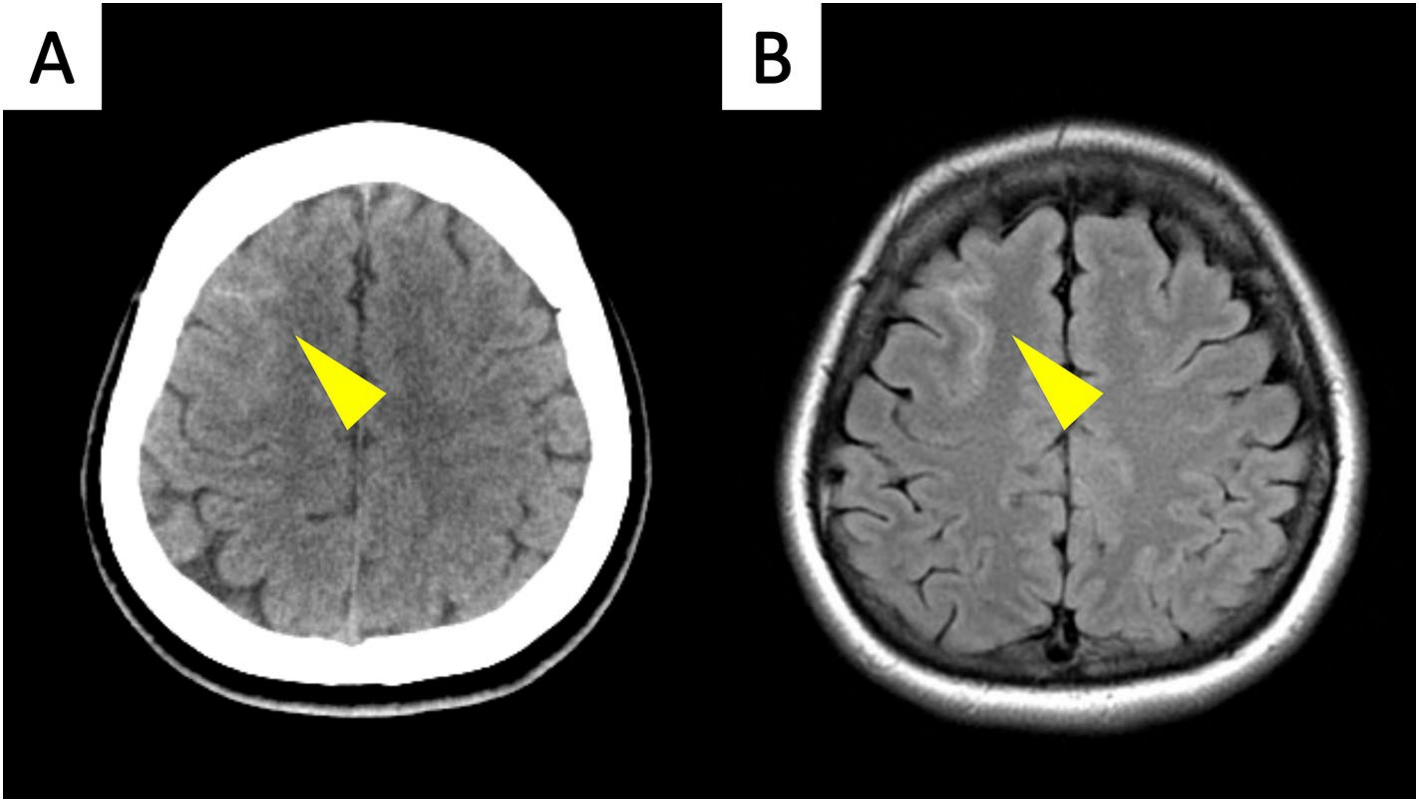
Diagnostic Imaging of Subarachnoid Hemorrhage. (**A**) Brain CT revealing a right parietal convexity subarachnoid hemorrhage (arrowhead). (**B**) T2-weighted FLAIR MR image obtained on admission showing high signal intensity in the right parietal convexity (arrowhead)

**Fig. 2:**
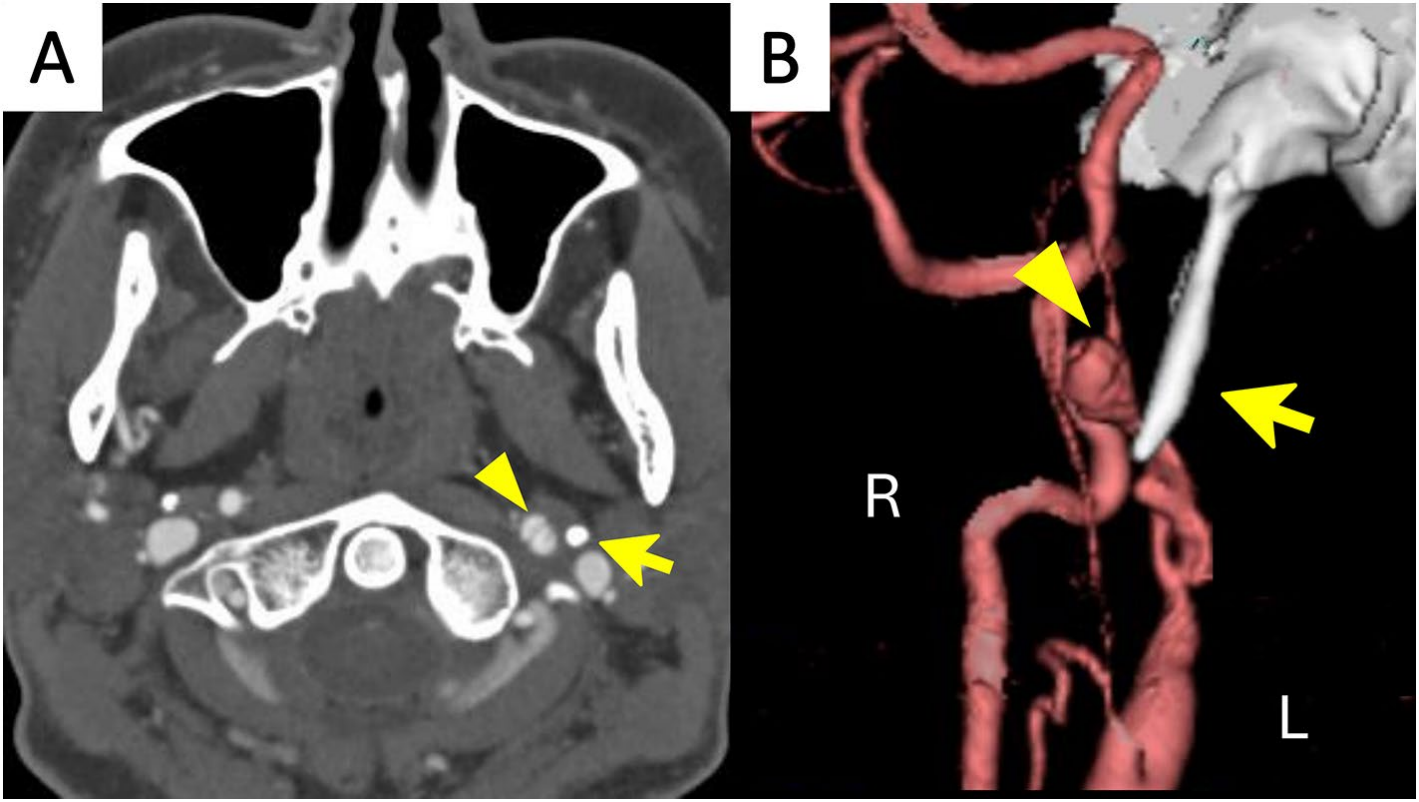
Computed tomography angiography (CTA) performed on the day of admission showing dissection of the left internal carotid artery with intimal flap (arrowhead) and an elongated styloid process (arrow). Original image (**A**) and 3D reconstructed image (**B**)

Brain computed tomography (CT) revealed SAH in the right parietal convexity (Fig. 1A). Subsequent cranial magnetic resonance imaging (MRI) using a T2-weighted fluid-attenuated inversion recovery (FLAIR) sequence confirmed the presence of cSAH (Fig. 1B). Diffusion-weighted imaging (DWI) and T2*-weighted imaging showed no discernible abnormalities. Notably, there was no evidence of PRES, cerebral amyloid angiopathy, or an underlying mass. Following the initial findings, computed tomography angiography (CTA) revealed dissection of the left ICA as well as an ESP measuring 30.1 mm on the left side and 27.5 mm on the right side. The left SP was located only 1.4 mm from the dissected artery (Fig. 2A and B). The patient was admitted to the hospital for further observation with the diagnosis of left ICAD accompanied by contralateral cSAH.

## Course of illness

On admission, the intensity of the headache was approximately 4/10 on the NRS. The headache gradually subsided, though the slight pain during body movement was prolonged. The headache fully resolved after approximately 14 days. The patient’s systolic blood pressure remained in the range of 120–140 mmHg without intervention. No ischemic symptoms were observed.

As cSAH is a potential complication of RCVS, we performed an MRA or CTA scan every three days. However, no indications of vasoconstriction were detected. Digital subtraction angiography (DSA) was performed on the fourteenth day of hospitalization and revealed the absence of vasospasm or shunt disease. Consequently, the patient was discharged without symptoms on the sixteenth day of hospitalization.

**Fig. 3:**
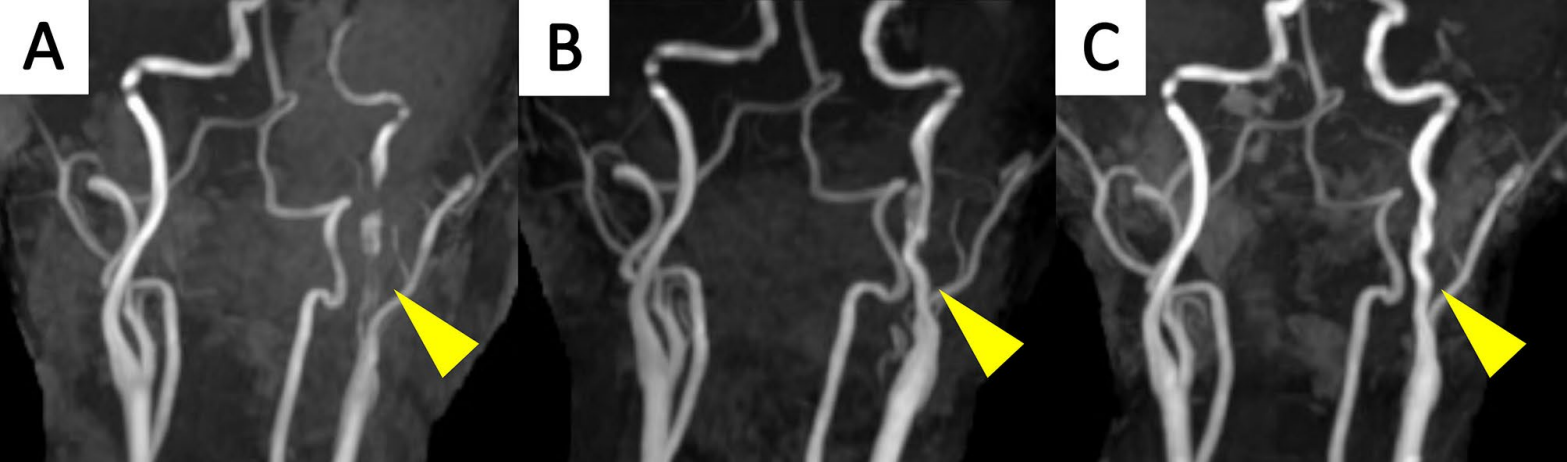
Longitudinal Observations of Left Internal Carotid Artery Dissection. (**A**) Magnetic resonance angiography (MRA) conducted on admission revealing dissection of the left internal carotid artery (arrowhead). Follow-up MRA images at 2 months (**B**) and 6 months (**C**) demonstrate progression toward resolution with conservative observation (arrowhead). The enlarged false lumen spontaneously regressed over time

We discussed our findings with the patient for further preventive therapeutic decision-making, including surgery and carotid stenting. She was reluctant to undergo interventional therapy and was managed conservatively without antithrombotic medication. Following outpatient observation, the left ICA lumen showed considerable improvement from the initial severe stenosis, though mild tortuosity persisted (Fig. 3A–C). The patient has remained recurrence-free for two and a half years. During this period, biannual MRA scans showed initial improvement in the first six months and no changes thereafter.

## Discussion and conclusions

Here, we described a case of ICAD attributed to an ESP concomitant with contralateral cSAH. This case is important for two distinct reasons. First, the co-occurrence of extracranial carotid artery dissection with contralateral cSAH is exceedingly rare. Second, the underlying cause is suggestive of Eagle syndrome, which introduces a unique aspect to the etiology.

## Etiology

Severe stenosis or occlusion of the intracranial or extracranial carotid arteries can cause cSAH [13, 14], typically occurring ipsilateral to the cSAH. Patients with contralateral involvement are extremely rare. While a few cases of ischemic stroke with contralateral cSAH have been reported [9], this patient exhibited no signs of cerebral infarction.

Initially, we considered the possibility of RCVS as a mediating factor between left-sided ICAD and right-sided cSAH. However, multimodal imaging failed to reveal any evidence supporting this hypothesis. Thus, we speculate that the rapid hemodynamic changes caused by the ICAD itself may lead to contralateral cSAH. Although there were no cerebral infarctions or ischemic symptoms, acute vascular diameter reduction in the main trunk due to dissection could prompt rapid dilation of the peripheral contralateral vessels as a compensatory mechanism. The collapse of vulnerable small vessels, which are incapable of withstanding this rapid burden, may lead to cSAH [15]. Another hypothesis is that a microthrombus originating at the dissection site may disperse contralaterally through the anterior communicating artery, precipitating tissue necrosis and microvascular rupture. However, this hypothesis was not substantiated in this patient, as DSA did not reveal blood flow from the left ICA to the contralateral side through the anterior communicating artery.

We believe that the etiology of ICAD in this patient may be associated with the identified ESP. Carotid artery dissection induced by ESP is recognized as the stylo-carotid subtype of Eagle syndrome [16]. The length of the SP is suggested to be a risk factor for cervical ICAD; an SP is considered elongated when it exceeds 30 mm [10, 12]. Additionally, a short distance between the SP and ICA is reportedly a considerable risk factor for ICAD [17]. The patient exhibited an SP measuring 30.1 mm, with a distance to the ICA of 1.4 mm. Given the absence of any apparent alternative causes, we concluded that the diagnosis was ICAD due to Eagle syndrome. In summary, we speculate that the ESP triggered ICAD, and the compensatory response to the rapid hemodynamic changes induced by the ICAD may have resulted in the cascading occurrence of contralateral cSAH.

## Treatment

As the patient had no symptoms other than pain, we opted for conservative treatment. Although antithrombotic treatment is generally recommended for patients with cervical dissection [18], there is a lack of consensus regarding its application when accompanied by cSAH. Thus, in this case, we refrained from introducing antithrombotic agents; this decision did not result in poorer outcomes for the patient.

There is no established evidence regarding the management of ICADs secondary to ESPs. Surgical intervention, including styloidectomy and carotid arterial stenting, can potentially prevent recurrence; however, the efficacy of these procedures and the optimal approach remain undefined. Recent literature reviews have shown that styloidectomy can effectively prevent the recurrence of Eagle syndrome without a considerable risk of complications [19, 20]. Hayashi et al. reported that 25% of patients receiving conservative treatment experienced recurrent ischemic events, in contrast to 0% of those who underwent styloidectomy, suggesting that surgical intervention is preferable [19]. Importantly, however, the cases reported in the literature may be biased toward those with favorable postoperative outcomes. Conservative treatment has been generally considered beneficial for ICAD management [21, 22], potentially including cases with ESP. It is unlikely that the length of the SP and its distance from the dissected ICA have been consistently measured in all patients with ICAD. There may be an unignorable number of ICAD patients with unrecognized ESP, making it difficult to draw accurate prognostic comparisons between conservative and surgical management. These limitations make it an insufficient basis for uniform recommendation of surgical intervention.

Another crucial consideration is that prophylactic surgery generally requires more meticulous risk management than curative surgery does. Surgical treatment carries a small but certain risk, including possible infection of deep neck spaces, injury to major vessels, facial nerve palsy (with an external approach), and postoperative discomfort [23]. As with any surgical procedure, it is imperative to thoroughly discuss its potential risks and benefits with the patient. Given her current preference to avoid surgical treatment for prevention, we opted to conservatively follow-up the patient. To accurately assess treatment outcomes, it is essential to compare groups recommended for surgical intervention with those not recommended, through well-designed prospective studies. Future research should prioritize the development of a standard treatment strategy for this condition.

## Conclusion

It is essential to consider ESP as a possible contributing factor in patients with carotid artery dissection as well as to assess the complications of cSAH. The optimal method for preventing recurrent cervical artery dissection due to Eagle syndrome is controversial; however, conservative management can be a viable option.

## Data Availability

No datasets were generated or analysed during the current study.

## Abbreviations

cSAH: convexity subarachnoid hemorrhage
CT: computed tomography
CTA: CT angiography
DSA: digital subtraction angiography
DWI: diffusion-weighted imaging
ESP: elongated styloid process
FLAIR: fluid-attenuated inversion recovery
ICA: Internal carotid artery
ICAD: Internal carotid artery dissection
MRA: magnetic resonance angiography
MRI: magnetic resonance imaging
PRES: posterior reversible leukoencephalopathy syndrome
RCVS: reversible cerebral vasoconstriction syndrome
SAH: subarachnoid hemorrhage
SP: styloid process
